# Peri-implant bone changes after using platelet-rich fibrin scaffold among Indians

**DOI:** 10.6026/973206300200600

**Published:** 2024-06-30

**Authors:** Abhishek Balani, Jaishree Tandon, Vinay Kharsan, Abhishek Karan, Heena Mazhar, Arunima Awasthy

**Affiliations:** 1 Department of Oral and Maxillofacial Surgery, New Horizon Dental College and Research Institute, Sakri, Bilaspur, Chhattisgarh, India

**Keywords:** Dental implants, PRF scaffold, bone loss

## Abstract

Bone transplant with osteopromotive elements - such as herbal extracts - that promote the creation of new boneis of interest to dentists. Hence, we compared the bone loss around dental implants while placing platelet rich fibrin (PRF) scaffold alone and PRF scaffold with simvastatin (SIM) and PRF scaffold with Moringaoleifera (MO). There were thirty six patients total. A total of 36 implants, or twelve implants in all three categories, were the estimated sample size. Category 1: PRF scaffolds alone. Category 2: PRF scaffolds with SIM. Category 3: PRF scaffolds with MO. Alteration in the bones were measured with CBCT. It was observed that there was decreased loss of crestal bone in PRF+ SIM and PRF+MO as compared to PRF alone. The use of herbal osteopromotive agents like simvastitin and Moringaoleifera along with PRF scaffolds can be effective in reducing bone loss around dental implants.

## Background:

Nowadays, one well-researched and validated course of rehabilitation for edentulous mandible is dental implant placement [1,2]. Bone alterations underlying an osseointegrated dental implant are thought to be a significant predictor of the implant's longevity, for a long time future outcomes, and overall success [3,4]. Therefore, the goal of implantologists is to minimize the destruction of bone surrounding an implant in the mouth [5, 6]. Any minor defect that cannot repair on its own over the course of the research study or the animal's lifespan is known as a critical-sized defect [7,8]. As mastication is significantly impacted by bone abnormalities, and occasionally aesthetics as well, delayed regeneration may be a therapeutic challenge [9,10]. Combining bone transplant with osteopromotive elements - such as herbal extracts - that promote the creation of new bone is one way to solve this issue [11,12]. The ease of application and cost-effectiveness of pharmacological approaches when paired with additional grafting ingredients have attracted a lot of attention [6, 8-9]. During the 1990s, simvastatin (SIM) and other pharmacological agents has been the subject of substantial research about their osteopromotive properties [10, 11, 12,13]. The hypercholesterolemic medication SIM lowers the level of cholesterol in blood and other intermediates by reversibly blocking the HMG-CoA reductase enzymes in the mevalonate cascade [9-12]. The pleiotropic consequences of SIM on the breakdown of bone are mostly linked to enhanced expression of the BMP-2 as well as vascular endothelial growth factor genes, which promote the development of osteoblastic cells [13, 15, 16]. By inhibiting the conjugation of precursors to osteoclast and lowering the production of TRAP along with cathepsin K, SIM has also been shown to decrease the breakdown of bones by reducing the total amount of active osteoclasts [8-11]. Therefore, in the realm of dentistry, SIM is now being studied to investigate its different methodology for working on bone in order to repair alveolar bone abnormalities and stop peri-implant loss of bone [12-15]. Various flavonoids found in Moringaoleifera (MO) leaves have the ability to cause mesenchymal stem cells produced from bone marrow to differentiate into osteoblasts [16, 17, 18, 19]. By eliminating free radicals, flavonoids shield cells against cellular damage caused by oxidative stress [17, 18, 19, 20, 21]. Many human trials have been conducted on moringaoleifera as a potential oral care product. One research investigation was carried out to evaluate the impact of leaf extract from Moringaoleifera on individuals who have early childhood caries. It was discovered that gargling using leaf extract from Moringaoleifera significantly reduces the production of plaque. [9- 18]. A biological scaffold made from human blood called platelet-rich fibrin (PRF) is found in a platelet concentrate that is acquired through centrifuge centrifugation [4-11]. It is extensively utilized in medicine and dentistry to promote regeneration of tissues and post-surgery recovery. A consequence of fibrin meshwork that has numerous growth agents is platelet-rich plasma [19, 22- 23]. It has been demonstrated that local PRF administration improves the healing process of critical-sized bone lesions and increases the regeneration of bones in experimental animals [24, 25, 26]. Therefore, it is of interest to compare the bone loss around dental implants while placing PRF scaffold alone and PRF scaffold with SIM and PRF scaffold with MO.

## Methods and Materials:

## Patient enrolment:

There were thirty six patients total. A total of 36 implants, or twelve implants in all three categories, were the estimated sample size. All the study participants were males between the age group of 45 years and 60 years,

Category 1: PRF alone

Category 2: PRF with SIM

Category 3: PRF with MO

## Criteria for inclusion and exclusion:

Patient selection was done using the outlined criteria for inclusion and criteria for exclusion in order to reduce the hazards associated with the present research, which include edema, pain following surgery, and ulcers following insertion of prosthesis.

## Qualifications for inclusion:

The study comprised patients whose partial dentition (a Kennedy class I mandible) had been recommended for restoration with implant prostheses, or who had undergone surgery or extractions at least six months prior.

Cone-beam computed tomography (CBCT) was used to assess bone density, and only patients with D3 bone were included in the study to maintain uniformity.

## Criteria for exclusion:

[1] Individuals who had severe attrition.

[2] Parafunctional uncontrollable habits.

[3] History of temporomandibular joint dysfunction.

[4] History of systemic medical conditions influencing bone condition or resorption.

[5] Patients receiving radiation therapy or chemotherapy.

[6] Those who smoke extensively.

[7] Vulnerable populations, such as patients with psychological disorders.

[8] Interviews were conducted in private settings with sufficient protection for patients' privacy. Furthermore, participants who made the decision to leave the research could continue get their regular care at the hospital.

[9] Each of the chosen individuals had a Kennedy class I condition and had experienced one to three years of incomplete dentition.

[10] The identical manufacturing procedures were used to create detachable partial dentures that were maintained by implants for each patient. Alterations in the bones were measured with CBCT.

## Prosthetic operations:

For every patient, Kennedy class I partial dentures were created. Initial impressions of the maxilla and mandible were taken utilizing alginate in stock trays, and final impressions were taken using an intermediate consistency rubber base in custom trays. To register jaw connection, bite blocks were built on the master casts. The artificial teeth were then fitted onto master casts that were positioned on a semi-adjustable articulator. The goal of preventing occlusal interactions in lateral excursions was to safeguard the implants. The patients' mouths were used for the waxed denture try-in, which was followed by process of flasking and processing into heat-cured acrylic resin. Before the denture was completed, the laboratory remounted it and made the required occlusal corrections.

## Procedures involving surgery:

To reduce the danger of infection, strict measures for infection control were implemented during every surgery. Every patient had a single stage of implant surgery, including the instantaneous installation of the partial prosthetic denture on the same day of implantation. After the patient's blood was removed in five milliliters, the sample was centrifuged for twelve minutes at a speed of three thousand rotations per minute (rpm). Subsequently, it separated into three layers: cellular plasma with a cream hue, a red section at the bottom that held red blood cells, and an intermediate layer that held the fibrin clot. Using sterile forceps, the intermediate component was extracted from the uppermost straw-coloured layer and transferred into a sterile Petri plate. In category 2, the osteotomy was filled with a manually prepared mixture of PRF and 1.2 mg SIM powder after implant insertion. In category 3, the osteotomy was filled with a manually mixed mixture of PRF and 1.2 mg MO powder after implant insertion. Every patient with an implant on their right side was assigned to the PRF group, while every patient with an implant on their left side was assigned to the SIM/PRF group. Throughout this investigation, all therapy procedures and follow-up were handled by the lead investigator and additional researchers. By keeping in constant communication with the participants and scheduling follow-up appointments on a monthly basis, the lead investigator was able to evaluate and document any adverse events.

## Assessment of skeletal modifications:

Utilizing a CBCT, the groups' respective implant sites' surrounding bone changes were assessed. At 0 months,3 months,6 months, and 12 months following implant implantation, CBCT was performed. Each implant's distal, buccal, mesial, lingual regions were measured, and a mean was determined using the data.

## An assessment of the stability of implants:

On the first day of installation and three months following the procedure, the Osstell device was used to assess the stability of the implant.

## Statistical analysis:

The mean ± standard deviation is used to present the data. A two-way analysis of variance was used to compare the outcomes between the groups at various time points, and a post hoc Bonferroni test was then performed. An unpaired t-test was used to compare stability and total bone changes between the two groups at the conclusion of the study. The software GraphPad Prism version 7.00 (GraphPad Software, San Diego, CA) was used for all experiments and calculations. A statistical significance threshold of P < 0.05 was applied.

## Results:

It was observed that there was decreased loss of crestal bone in PRF+ SIM and PRF+MO as compared to PRF alone. The findings were statistically significant. The mean values of crestal bone loss decreased in all categories as the time progressed. The findings were significant statistically. It was also noticed that that the bone loss was comparable in PRF+ SIM and PRF+ MO (Table 1). The implant stability was comparable in all three categories with more than 50% implant stability in each category (Table 2).

## Discussion:

According to previous research, using SIM locally rather than systemically has shown to be more effective in treating mandibular abnormalities. A study documented a 240% increase in bone density following local application of SIM for mandibular abnormalities [6-11]. On the other hand, another study examined how SIM affected closed defects such as distraction osteogenesis and found that the group of statins administered locally had a larger surface area of the analyzed bone [4-9]. Because the local delivery of SIM avoids the drug's systemic side effects and hepatic breakdown, it was chosen for the current investigation [15-19]. Prior in vitro research examined the osteopromotive impact of SIM. The specifics of osteoblast differentiation triggered by SIM were elucidated in a study which also found that SIM enhanced osteoblast survivability and maturation [11-17]. A study observed an increase in the dispersion of osteoprotegerin, a potent anti osteoclastic chemical substance, released from MSCs placed on TiO2 scaffolds [3-8]. A study also looked into how SIM affected the morphological transformation and maturation of osteoblasts precursors [4- 11]. Prior research using laboratory animals documented the impact of SIM in reducing bone resorption. In rats with osteoporosis and periodontitis, researchers examined the local and systemic effects of SIM on halting bone loss [2-9]. Their study's findings, which included histomorphometric and radiographic examination, demonstrated that the use of SIM increased the peak of the alveolar crest and prevented the loss of alveolar bone [11-18]. Furthermore, utilizing histological analysis, a study also reported data indicating decreased periodontal bone loss following subperiosteal SIM delivery in the jaw [15-19]. A recent study investigated the efficacy of systemic SIM injection as a treatment for hypercholesterolemia-induced alveolar bone loss, as well as potential mechanisms of action [11-17].The findings of the study demonstrated that SIM administration effectively reduced the transcription of RANKL mRNA, downregulated NF-κB production, and significantly attenuated the alveolar bone loss caused by hypercholesterolemia [10-17]. An in vitro investigation evaluated the free radical scavenging properties of the extract of Moringaoleifera leaves and found that it may have an anti-inflammatory antioxidant property [16-23]. By lowering the synthesis of Interleukin-6 (IL-6) chemical mediator, a pro-inflammatory cytokine produced by Porphyromonasgingivalis, Moringaoleifera has additionally been shown to have anti-inflammatory properties [13- 17]. Additionally, we discovered that the total number of osteoblasts associated with the defect - particularly in the proliferative stage - was markedly boosted by the incorporation of the extract of Moringaoleifera leaves to the alloplastic bone transplant. These findings are consistent with a study conducted earlier which discovered that saponins influence osteoblastic proliferation as well as differentiation through an in vitro osteogenic activity [18- 26]. While all of the flavonoids found in Moringaoleifera are vital for bone regeneration, kaempferol flavonoids have been shown to be particularly significant [12-18]. Osteoblast differentiation is induced by kaempferol activating estrogen receptors. Tannin content in dried moringa leaves ranges from 13.2 to 20.6 g/kg. This is very high tannin content. These tannins have the ability to suppress osteoclast differentiation, thereby which promotes the growth of new bone[7-15].The impact of the extract of Moringaoleifera leaves on how orthodontic teeth move following its application in tension zones was studied. In the locations where it was applied, it was found that the extract of Moringaoleifera leaves significantly enhanced the quantity of osteoblasts & diminished the proportion of osteoclasts [9- 17].

## Conclusion:

The use of herbal osteopromotive agents like Simvastitin and Moringaoleifera along with PRF scaffolds can be effective in reducing bone loss around dental implants.

## Figures and Tables

**Table 1:** Changes in crestal bone at different time intervals in different categories

**Variable**	**0-3 months**	**3-6 months**	**6-12 months**
PRF (Mean±SD) mm	0.64±0.055	0.46±0.057	0.33±0.044
PRF/SIM (Mean±SD) mm	0.43±0.032	0.32±0.039	0.27±0.045
PRF/MO(Mean±SD) mm	0.47±0.043	0.35±0.041	0.29±0.056
MD	0.324	0.1511	0.0691
SED	0.0169	0.0169	0.0269
P-value	<0.0001*	<0.0001*	0.0028*

**Table 2:** Implant stability at baseline and 3 months postoperatively

**Variable**	**0-3 months**	**3-6 months**
PRF (%)	59.4± 2.68	73.8±1.50
PRF/SIM (%)	61.1± 3.22	73.7±2.14
PRF/MO (%)	60.2± 1.14	74.8±2.14
P-value	0.5216	0.8121
